# Parental Warmth as a Moderator Between Family Conflict and the use of Corporal Punishment: Adolescents’ Perspectives

**DOI:** 10.1007/s40653-026-00867-6

**Published:** 2026-04-11

**Authors:** Natalia Valenzuela, Angela Trujillo, Martha Rocío González

**Affiliations:** Facultad de Ciencias del Comportamiento, University of La Sabana, Chía, Colombia

**Keywords:** Parental Warmth, Family Conflict, CP, Youth mental health

## Abstract

This study examined whether parental warmth moderates the relationship between family conflict and physical punishment from the perspective of Colombian adolescents. A sample of 3,279 adolescents (ages 10–19) from six municipalities in Cundinamarca completed the Communities That Care Youth Survey. Using multiple linear regression and moderation analysis, results revealed that higher parental warmth buffers the association between family conflict and the use of Physical punishment. Specifically, as parental warmth increases, the impact of family conflict on CP decreases. These findings highlight the protective role of warm parenting in mitigating negative family dynamics and have important implications for developing interventions and public policies to reduce harsh discipline in Colombia.

Parenting practices play a critical role in shaping children’s socioemotional and behavioral development (Hoeve et al., 2009; Smetana & Rote, 2019). Globally, an estimated 400 million children are exposed to violent discipline at home (Fondo de las Naciones Unidas para la Infancia [UNICEF], 2024), with nearly 60% of youth aged 2–14 experiencing CP regularly (UNICEF, 2018). In Colombia, research indicates a high prevalence ranging from 41% in rural areas (González et al., 2014) to 77% in recent national samples (Trujillo et al., 2020). Consequently, there is an urgent need to identify the family dynamics that sustain these practices despite current legal prohibitions (Cuartas et al., 2019a).

Although research suggests that CP aims to modify behavior rather than inflicting harm (Larzelere & Kuhn, 2005), a robust body of research demonstrates the detrimental inflicts on children’s development and well-being, as well as its ineffectiveness in promoting positive behavioral change (Gershoff & Grogan-Kaylor, 2016). Empirical evidence consistently associates CP with the development of externalizing behaviors in childhood (Wiggers & Paas, 2022; World Health Organization (WHO, 2024) and internalizing difficulties in adulthood (Ferguson, 2013; Hecker et al. 2016).

Parental use of CP is shaped by a range of family related factors, including parental backgrounds (Hughes et al., 2022), maternal attitudes (González et al., 2024; Rancheiro et al., 2023), sociodemographic characteristics, and family structure (González et al., 2014). Understanding these associations is essential to inform interventions aimed at reducing the prevalence of CP. Family conflict is one variable that has received comparatively less empirical attention in this context. Scientific evidence shows that the prevalence of CP is notably higher among families experiencing conflict, (Gershoff & Grogan-Kaylor, 2016; Hughes et al., 2022).

While conflict is a natural part of family life, unresolved conflict can have adverse consequences for all members involved. Family conflict is an interaction characterized by persistent tension or emotional dysregulation that reflects overall family functioning. While chronic conflict is associated with diminished emotional resources and a higher frequency of reactive disciplinary responses, protective factors such as parental warmth—the degree of affection and emotional support conveyed in everyday interactions (Rohner, 2004). —may moderate these associations (Cuartas et al., 2021; Font & Maguire-Jack, 2020; Gershoff & Grogan-Kaylor, 2016; Farrington & Malvaso, 2023; Lemos & Pérez, 2021; Parolin & Simonelli, 2016; Wang et al., 2022; Ward et al., 2020). Parental warmth encompasses both observable behaviors—such as physical affection, praise, and positive facial expressions—and a stable emotional orientation characterized by care, empathy, and respect for the child’s individuality (Rohner et al., 2020).

Prior research indicates that while warmth alone may be insufficient to account for the absence of CP in culturally normalized contexts, it functions as a relational context that can attenuate the link between family conflict and harsh discipline (Alampay & Alampay, 2019; Wiggers & Paas, 2022; Yang et al., 2023). Effective parental involvement—characterized by warm, responsive, and emotionally supportive interactions— is consistently associated with a lower frequency of CP and may serve as a relational context that modulates the link between family conflict and disciplinary practices ((Boullion et al., 2023; Cuartas et al., 2021; Wang et al., 2023; Yang et al., 2022). This interpretation aligns with Rohner’s interpersonal acceptance–rejection theory, which posits that warmth reflects a stable emotional orientation rather than a discrete parenting behavior. While some evidence suggests that the behavioral and emotional correlates of CP may be less severe when it occurs within a highly warm and supportive family climate, warmth does not eliminate developmental risks, as CP remains associated with elevated externalizing problems even in affectionate households (Deater-Deckard et al., 2006; Lansford et al., 2014). Consequently, parental warmth operates less as a direct deterrent and more as a conditional buffer (Pan et al., 2024), whose precise role in the relationship between family conflict and CP requires further empirical clarification within the Colombian context.

Other studies suggest that the moderating role of parental warmth is shaped by cultural context. In cultures where CP is viewed as normative, warmth may exert a buffering or mitigating influence. In contrast, in cultures where CP is socially disapproved, the coexistence of warmth and CP may create emotional dissonance, increasing children’s vulnerability to anxiety or guilt (Deater-Deckard et al., 2006; Lansford et al., 2014; Lee et al., 2013). In highly conflictual family environments, the protective effects of warmth appear to weaken. When family interactions are dominated by tension, yelling, or threats, the influence of warmth tends to diminish or become inconsistent (Wang et al., 2018). Furthermore, emerging evidence indicates that warmth cannot fully offset the cumulative impact of family conflict. Persistent conflict is associated with a decrese in warmth’s protective value, rendering it inconsistent or even confusing for the child. Consequently, sporadic expressions of affection cannot compensate for the enduring effects of family conflict and punitive (Wiggers & Paas, 2022).

Taken together, the current body of evidence underscores the need for further research examining family conflict as a salient risk factor, while highlighting the close association between CP and family conflict. Previous findings suggest that parental warmth may serve as a weak or partial moderator, but its role appears inconsistent and culturally dependent.

Therefore, the present study examines the moderating effect of parental warmth on the association between family conflict and the use of CP from the perspective of Colombian adolescents. Accordingly, this study provides empirical evidence to clarify these interrelations while identifying how parental warmth may modulate the link between conflict and disciplinary practices (Fig. 1).

**Fig. 1:**
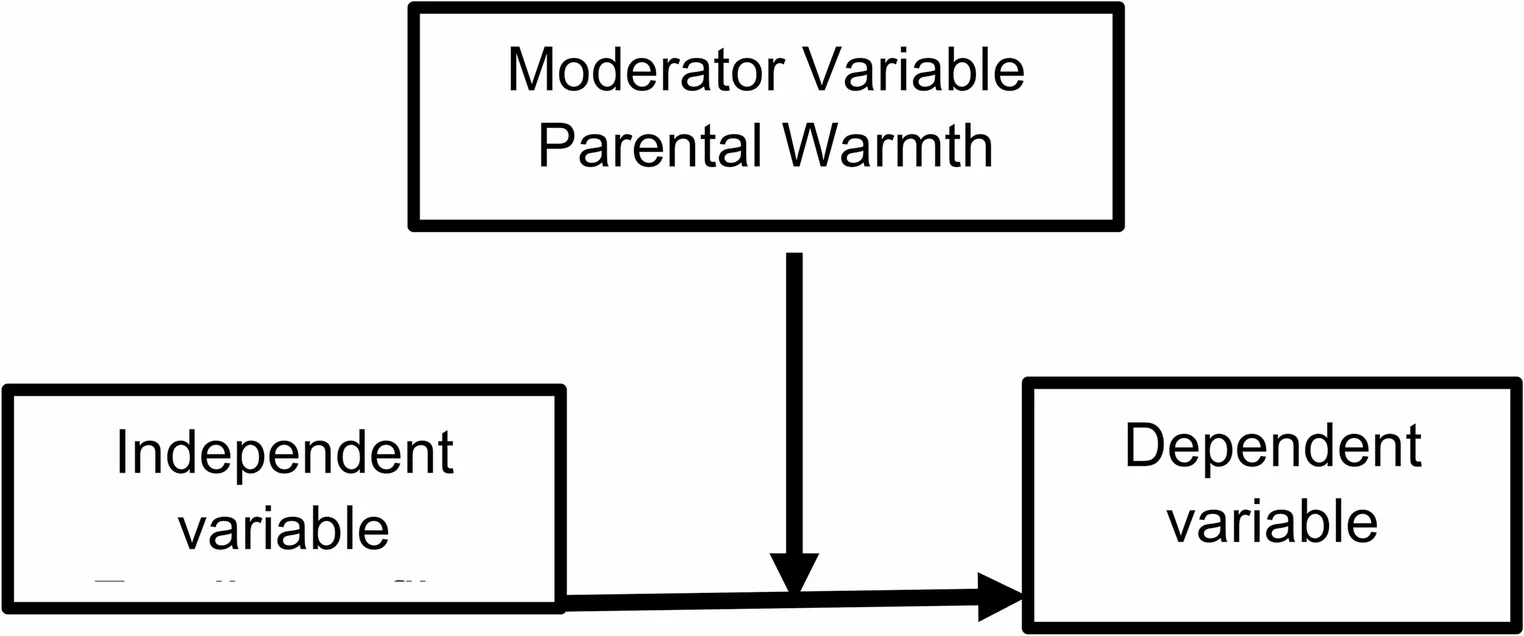
Moderation Model

## Hypotheses

- **H1:** Higher levels of family conflict will be associated with increased use of CP, as perceived by adolescents in Cundinamarca, Colombia.
- **H2:** Greater parental warmth will be associated with lower reported use of CP among adolescents.
- **H3:** Parental warmth will moderate the relationship between family conflict and CP, such that the association between family conflict and CP will be weaker among families characterized by higher parental warmth.

## Method

### Study Design

This study utilized a cross-sectional, non-experimental correlational design. Data were collected at a single point in time to assess the relationships between family conflict, parental warmth, and the use of CP among adolescents. A moderation analysis using multiple linear regression will be employed to assess how an independent variable (family conflict) predicts a dependent variable (use of CP), considering the influence of a third moderating variable (parental warmth) that affects the strength and/or direction of this relationship (Hayes, 2017). Following the approach proposed by Fairchild and MacKinnon (2009), moderation analysis relies on multiple regression models to evaluate both the individual impact of predictor variables (family conflict and parental warmth) on the dependent variable, as well as the interaction between them. When the interaction is significant, the effect of family conflict on the use of CP cannot be interpreted in isolation, as its magnitude and direction depend on levels of parental warmth. Linear regressions were utilized to examine the relationships between the variables and determine the moderating effect of parental warmth, seeking to identify the extent to which parental warmth modulates the relationship between family conflict and the use of CP from the youth’s perspective.

### Participants

Data for this research were obtained through the social outreach project of the Department of anonymized at anonymized, titled " anonymized (project number PSIPHD-4–2023). The sample included 3,279 adolescents attending public educational institutions across six municipalities in the department of anonymized, Colombia. These municipalities included both urban and rural settings geographically located near the capital city of Bogotá. The participating public schools primarily serve students form families classified within low socioeconomic strata (SES) (generally levels 1 and 2 as defined by Colombia’s official socioeconomic stratification system), providing a sample highly representative of vulnerable and low-to-middle income youth in the region. The adolescents belonged to low and middle socioeconomic levels.

The average age of participants was 14.30 years (SD = 1.64), with an age range from 10 to 19 years of the total sample, 53.1% were female (*n* = 1,740), and 46.9% were male (*n* = 1,539). The sample was selected to ensure its representativeness of the school population in the six municipalities included in the study. The distribution by municipality was as follows: municipality 1 (*n* = 835; 25.4%), municipality 2 (*n* = 808; 24.6%), municipality 3 (*n* = 489; 14.9%), municipality 4 (*n* = 419; 12.7%), municipality 5 (*n* = 412; 12.5%), and municipality 6 (*n* = 316; 9.6%).

Regarding the academic distribution of the participants, 9.3% were in sixth grade, 19.7% in seventh, 24% in eighth, 25% in ninth, 16.1% in tenth, and 5.7% in eleventh grade. The data collected included information about family conflict, parental warmth, and the use of CP by parents (Table 1).

**Table 1:** Sociodemographic Characteristics of the Adolescent Sample

Socio-demographic variable		*%*	*%*	*%*	*%*	*%*	*%*
**Sex**	Female	50.65	53.34	55.62	57.99	48.78	53.79
	Male	49.34	56.65	44.37	42.00	51.21	46.20
**Age**	10	0.12	0.00	0.00	0.00	0.12	0.63
	11	2.27	0.12	5.72	0.95	2.27	6.01
	12	13.53	0.99	17.38	19.80	13.53	5.38
	**13**	**22.51**	**13.49**	**21.67**	**21.48**	**22.51**	**6.32**
	**14**	**22.27**	**23.88**	**18.40**	**30.78**	**22.27**	**12.34**
	**15**	**22.15**	**28.58**	**20.65**	**16.22**	**22.15**	**23.10**
	16	12.57	20.17	9.20	8.83	12.57	23.41
	17	3.71	8.78	5.93	1.90	3.71	15.82
	18	0.59	3.58	0.61	0.00	0.59	5.38
	19	0.24	0.37	0.40	0.00	0.24	1.58
**Grade**	Sixth	1.55	0.74	24.13	0.23	27.91	17.40
	Seventh	32.81	0.61	25.15	33.65	21.60	4.74
	Eighth	21.79	37.50	17.99	32.69	18.44	0.31
	Nineth	22.63	33.66	19.83	33.41	15.04	18.98
	Tenth	21.19	25.50	0.00	0.00	16.99	21.58
	Eleventh	0.	2.97	12.88	0.00	0.12	31.96

### Procedure

The sample was recruited through coordination with the health and education secretariats of various municipalities in anonymized. The project was presented to these local authorities to invite their participation and to engage public educational institutions in the data collection process. After securing the municipalities’ consent, the lead researcher conducted training sessions for the designated professionals appointed by the secretariats. These sessions provided comprehensive guidance on administering the research instruments and obtaining informed consent from the legal guardians of the adolescent participants. This process ensured that participation was voluntary and confidential, adhering to ethical research standards. Data collection was conducted during the school day, specifically in computer rooms, with each group of students in sessions lasting approximately one hour. Before responding to the questionnaire, each adolescent signed an informed assent, which included the following statement: “I agree to participate in this project, and I understand that my responses will not be identified by anyone from the school or by my parents. By participating, I help develop strategies to prevent risk behaviors among adolescents.” The options for this statement were “Yes” or “No.” The questionnaire was administered electronically in 34 educational institutions from the six municipalities of anonymized. Trained professionals in administering the Communities That Care Youth Survey (CTC-YS) (Arthur et al., 2002) were responsible for its implementation in schools. The digital version of the questionnaire was configured to ensure that all questions were mandatory, preventing missing data generation. To maintain confidentiality, each participant was assigned a unique code, and responses were recorded in an Excel file.

### Instruments

Data was collected using the Communities That Care Youth Survey (CTC-YS) (Arthur et al., 2002), designed to evaluate risk and protective factors among adolescents. This questionnaire identifies the levels of exposure to various variables associated with substance use and disruptive behaviors, comprising 165 items distributed over 34 factors, with internal consistency ranging from 0.59 to 0.88. The CTC-YS has been validated in Colombian adolescent populations (Obando et al., 2014). In this research, the Spanish version of the CTC-YS (Arthur et al., 2002) was used to evaluate three key dimensions: family conflict, parental warmth, and the use of CP.

Family Conflict was measured using a six-item subscale derived from the Communities That Care Youth Survey (CTC-YS) (Arthur et al., 2002). Adolescents reported on this variable using language that refers to the family system generally (e.g., “In my family…” or “My parents…”) This measure assesses the adolescent’s perception of the conflictual environment within the family system, encompassing multiple dimensions of conflict. These dimensions include the frequency of chronic/unresolved conflict (e.g., *“In your family*,* do you argue about the same things over and over?”*), verbal/emotional conflict (e.g., *“Do people in your family shout or insult each other?”*), marital/parental conflict, and severe/physical conflict. Items were rated on a scale (e.g., 1 = *Never* to 4 = *Often*). The composite variable was created by calculating the mean score of the items, with higher scores indicating higher perceived family conflict (α = 0.7609) as proposed by the authors of the survey.

Parental Warmth was assessed using a five-item subscale that captures the adolescent’s perception of emotional closeness and support from both maternal and paternal figures (e.g., ‘I feel close to my mother,’ ‘I share my thoughts and feelings with my mother’ or the equivalent for the father: ‘I feel close to my father,’ ‘I share my thoughts and feelings with my father’). While individual items distinguish between caregivers, a single composite mean score was calculated to represent the generalized affective climate of the family unit. This approach is theoretically grounded in the concept of the family as an interdependent emotional system, where the net availability of warmth serves as a primary protective resource for the adolescent. Conceptually, this composite score reflects the average emotional availability in the household; thus, a high score indicates a consistently warm environment, while a moderate score may represent a compensatory dynamic where one caregiver’s warmth mitigates the relative coldness of another. For both parents, the instrument includes an option for adolescents to indicate if a caregiver is absent (e.g., ‘I don’t have a mother/father’). In such cases, the composite score was calculated based on the available caregiver to accurately reflect the active emotional support system accessible to the adolescent, a method that aligns with prevention research standards using the CTC-YS to evaluate aggregate protective factors.

The dependent variable, Use of CP, was measured using a single item *“My parents use CP to correct me (slapping*,* spankings…”* Participants responded on a 4-point Likert scale (1 = never to 4 = often). The decision to use a single-item measure was a deliberate methodological and ethical choice. Following the enactment of Law 2089 of 2021 in Colombia, which legally prohibits CP, this behavior has become highly sensitive. A single, non-accusatory item was selected to maximize participant confidentiality and minimize the risk of psychological distress or re-victimization, ensuring compliance with local ethical protocols. While single items are common in large-scale community surveys to reduce respondent burden, we acknowledge that this may impact the ability to calculate internal consistency.

For data analysis, Pearson product-moment correlations were initially computed to examine the zero-order associations among Family Conflict, Parental Warmth and CP (Preacher and Hayes, 2008). To test the hypothesized moderation, we conducted a moderated multiple regression analysis using Model 1 of the PROCESS macro (version 16.3) for SPSS (version 26) (Hayes, 2017). This procedure employed Ordinary Least Squares (OLS) regressions to estimate the effect of family conflict (independent variable) on the use of CP (dependent variable), with parental warmth serving as the moderating variable.

To ensure statistical transparency and mitigate potential multicollinearity between the main effects and the interaction term, both the predictor (Family Conflict) and the moderator (Parental Warmth) were mean-centered prior to the construction of the interaction term. The final model included three components entered simultaneously: (1) the main effect of Family Conflict; (2) the main effect of Parental Warmth; and (3) the interaction term (Family Conflict X Parental Warmth). Model estimation employed bias-corrected bootstrapping with 5,000 resamples to compute robust standard errors and 95% confidence intervals (CIs) for the direct effects and the interaction term.

## Results

The results are presented in three parts: descriptive statistics, correlations among the measured variables, and the moderated multiple regression analysis testing the study’s central hypothesis.

Descriptive statistics: Table 2 presents the mean (M) and standard deviation (SD) for the composite variables included in the proposed model. Family Conflict was observed at a low-to-moderate level (M = 1.65, SD 0.49). on a 1 to 4 scale, indicating that participants generally perceive a moderate frequency of conflict among caregivers. Parental warmth demonstrated a medium level of perceived closeness and support (M = 2.82, 0.57) Regarding the dependent variable, use of CP, the mean score was M = 2.02 (SD = 0.87), reflecting a low overall frequency of this disciplinary practice in the sample.

**Table 2:** Descriptive Statistics for the Variables

Variable	Mean	Standard Deviation
Family Conflict	1.65	0.49
Parental Warmth	2.82	0.57
Use of CP	2.02	0.87

Further analysis of use of CP indicated that 69,18% of participants reported experiencing CP at least once (comprising 43,06% occasionally, 19,51% frequently, and 6,61% regularly). The relatively high standard deviation reflects considerable variability in adolescents’ reports of this behavior.

Pearson correlation analyses revealed statistically significant associations among all study variables (Table 3). A positive and moderate association was found between Family Conflict and CP (*r* = 0.345, *p* < 0.01) suggesting that higher levels of family conflict are linked to an increased likelihood of parents employing CP as a disciplinary strategy.

**Table 3:** Pearson Correlation among Family Conflict, Parental Warmth, and Use of CP

Variable	Use of CP	Family Conflict	Parental Warmth
Use of CP	—	0.345**	−0.058**
Family Conflict	0.345**	—	−0.231**
Parental Warmth	−0.058**	−0.231**	—

*N* = 3279. *p* < 0.001 (two-tailed). **Correlation is significant at the 0.01 level (two-tailed)

Conversely, parental warmth demonstrated negative correlations with both CP (*r* = −0.058, *p* < 0.01) and family conflict (*r* = −0.231, *p* < 0.01). This indicates that greater perceived parental closeness and support are associated with lower levels of family conflict and reduced use of CP.

The moderated multiple regression analysis, conducted using the PROCESS macro, confirmed the study’s central hypothesis. The overall model was statistically significant, F (2, 2960) = 196.52, *p* < 0.001, accounting for 11.7% of the variance in the use of CP (R² = 0.117). While family conflict was a significant predictor, the interaction term (Family Conflict X Parental Warmth) was the key finding, being statistically significant (β = −0.152, t = −3,444 *p* = 0.001) and explaining an additional 0,4% of the variance (**Δ*R***²): 0.4\% (F_change_ (1, 2959) = 11.862, *p* = 0.001).

To ensure the robustness of the moderation, simple slopes were analyzed at three levels of warmth (Table 4). Conflict remained a significant predictor across all levels, but the effect size decreased as warmth increased, a trend confirmed by the non-overlapping $95\%$ confidence intervals:

- Low warmth (−1SD): β = 0.684$, 95% CI [0.612, 0.756].
- Mean Warmth: β = 0.597, 95% CI [0.534, 0.661].
- High Warmth (+ 1 SD): β = 0.511$, 95% CI [0.423, 0.599].

**Table 4:** Conditional Effects of Family Conflict (Simple Slopes) on the Use of CP at Different Levels of Parental Warmth

Parental Warmth Level	Effect (β)	SE	t	*p*	95% CI
Low (−1 SD)	0.684	0.036	18.643	<,001	(0.612, 0.756)
Mean (M)	0.597	0.032	18.448	<,001	(0.534, 0.661)
High (+ 1SD)	0.511	0.045	11.375	<,001	(0.423, 0.599)

*N* = 3279. Conditional effects calculated at *+1*standard deviation from the mean

**Fig. 2:**
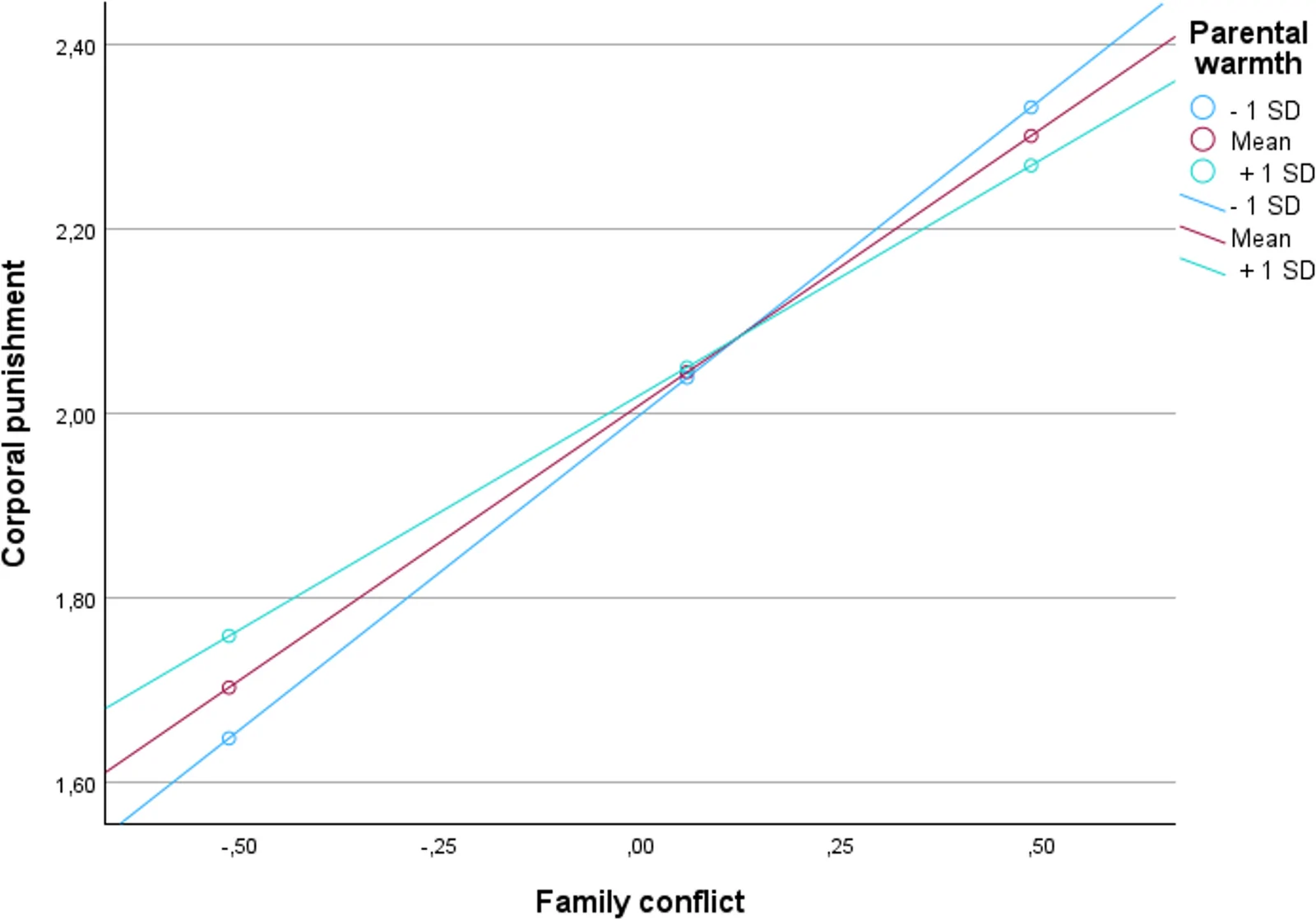
Interaction Effect of Parental Warmth on the Relationship between Family Conflict and the Use of Corporal Punishment Note: N = 3279. The lines represent the association between family conflict and the use of CP at three levels of parental warmth: low (−1 SD), mean, and high (+ 1 SD). Values on the X-axis represent mean-centered scores for family conflict. The steeper slope for the low-warmth group indicates a stronger association between conflict

As illustrated in Fig. 2, the interaction plot visually confirms the buffering hypothesis. The steepest line represents adolescents with low parental warmth, indicating a stronger link between conflict and CP. In contrast, the line for high parental warmth is notably less steep, illustrating that emotional closeness attenuates the impact of family conflict.

## Discussion

The present study examined the moderating role of parental warmth in the association between family conflict and the use of physical punishment (CP), as perceived by adolescents in Cundinamarca, Colombia. By integrating adolescents’ perspectives, this research contributes novel empirical evidence to a growing body of literature that conceptualizes CP not as an isolated parenting behavior, but as a practice embedded within broader family dynamics characterized by stress, conflict, and relational quality (Cuartas et al., 2021; Gershoff & Grogan-Kaylor, 2016; Wiggers & Paas, 2022). Overall, the findings underscore the complex interplay between risk and protective factors in family systems and highlight parental warmth as a critical, albeit conditional, buffer against the use of coercive disciplinary practices.

### Family Conflict as a Risk Factor for Physical Punishment (H1)

Consistent with Hypothesis 1, higher levels of family conflict were positively and significantly associated with adolescents’ reports of CP. While the cross-sectional nature of this study precludes definitive causal pathways, these findings suggest that environments characterized by interpersonal tension frequently co-occur with harsher disciplinary strategies (Font & Maguire-Jack, 2020; Gershoff & Grogan-Kaylor, 2016; Hughes et al., 2022; Ward et al., 2020). From a family stress perspective (Deater-Deckard & Dodge, 2009), chronic conflict is associated with diminished emotional resources among caregivers, a state that correlates with a higher frequency of reactive, rather than regulated, responses to child behavior.

Importantly, the present findings support conceptualizations of family conflict as both a contextual and transactional correlate. Prior research suggests that conflict and CP may be bidirectionally related, where interpersonal tension and physical discipline co-occur within coercive cycles that reinforce maladaptive interaction patterns (Wiggers & Paas, 2022). In adolescence—a developmental period marked by increasing autonomy-seeking and heightened parent–child negotiations—these dynamics may become particularly salient (Fosco & Lippold, 2019). Thus, the strong association observed in this study reinforces the need to conceptualize CP within broader relational climates rather than as an isolated disciplinary choice.

Within the Colombian context, this finding is especially concerning. Exposure to structural violence, social inequality and armed conflict has historically shaped norms surrounding authority and discipline (Cuartas et al., 2019a). As such, family conflict may function as a proximal stressor that interacts with broader sociocultural conditions, increasing reliance on physical punishment even in the presence of legal prohibitions such as Law 2089 of 2021.

### Parental Warmth and its Direct Association With Physical Punishment (H2)

 Hypothesis 2 proposed that higher levels of parental warmth would be associated with lower reported use of CP. The correlational analyses partially supported this hypothesis, revealing a weak but significant negative association between parental warmth and CP. This finding is consistent with prior studies indicating that warm, emotionally responsive parenting co-occurs with lower baseline hostility and lower frequency of coercive disciplinary practices (Cuartas et al., 2021; Yang et al., 2022). Rather than a direct causal deterrent, warmth may represent a relational context where punitive strategies are less common.

However, the modest strength of this association warrants careful interpretation. As highlighted in previous research, parental warmth alone may be insufficient to account for the absence of CP, particularly in contexts where physical discipline is culturally normalized or embedded in broader patterns of family stress (Gershoff & Grogan-Kaylor, 2016; Pan et al., 2024). Indeed, several studies have demonstrated that even within affectionate family climates, CP remains associated with adverse child outcomes, such as a higher frequency of externalizing behaviors (Lansford et al., 2014; Pan et al., 2024). This underscores the importance of viewing warmth not as a direct deterrent, but as one of several interrelated relational factors.

Thus, the present findings support the view that parental warmth operates less as a direct protective factor and more as a relational context that modulates the association between other risk processes—such as family conflict—unfold. This interpretation aligns with Rohner’s interpersonal acceptance–rejection theory (Rohner, 2004; Rohner et al., 2020), which emphasizes that warmth reflects a broader emotional orientation rather than a discrete parenting behavior. Within this framework, warmth is viewed as a stable emotional climate that correlates with the nature of interpersonal interactions rather than a simple reaction to specific behaviors.

### The Moderating Role of Parental Warmth (H3)

The central contribution of this study lies in its support for Hypothesis 3: parental warmth functioned as a significant moderator of the association between family conflict and CP. Specifically, the positive relationship between family conflict and CP was significantly more evident among adolescents who reported low levels of parental warmth. In contrast, in families characterized by higher levels of warmth, this association was lower in magnitude, suggesting that emotional closeness represents a relational context where conflict and physical discipline are less strongly linked.

This finding provides empirical support for theoretical models suggesting that warmth represents a relational context characterized by lower levels of spillover between family conflict and harsh disciplinary practices (Alampay & Alampay, 2019; Boullion et al., 2023). In families where adolescents perceive high emotional closeness and acceptance, conflict and punitive responses are less strongly linked, even in the presence of frequent disagreements. This pattern aligns with the principles of Trauma-Informed Care (TIC), which suggest that a warm emotional climate may be associated with lower threat-perception in children, potentially disrupting the co-occurrence of interpersonal hostility and physical aggression.

A nuanced finding in the moderated regression analysis was that while parental warmth was a significant moderator, it did not demonstrate a significant direct effect on CP. This suggests that parental warmth is not an independent ‘deterrent’ that simply lowers the frequency of punishment in all cases. Instead, its value is conditional; it functions by ‘de-coupling’ the strong association between family conflict and CP observed in low-warmth households. This indicates that the presence of an affectionate emotional climate changes the nature of how caregivers respond to family stress, preventing interpersonal tension from automatically translating into physical discipline. (Deater-Deckard et al., 2006; Lansford et al., 2014; Wiggers & Paas, 2022). In high-conflict households, persistent exposure to hostility and emotional dysregulation correlates with a diminished protective value of warmth, where affectionate behaviors may be perceived as inconsistent or confusing by adolescents (Wang et al., 2018). These findings align with the principles of trauma-informed care (TIC), suggesting that a warm emotional climate is associated with lower activation of ‘threat-detection’ patterns often observed in youth in high-conflict environments. These findings provide specific empirical support for the Child-Directed Interaction (CDI) phase of Parent-Child Interaction Therapy (PCIT) (Gurwitch, Messer, Funderburk & Warner, 2025). CDI focuses on strengthening the attachment bond through the ‘PRIDE’ skills—Praise, Reflection, Imitation, Description, and Enjoyment—to build the exact ‘warmth’ moderator identified in this study. By establishing a secure and responsive relational context during this initial phase, the CDI skills may create a ‘buffer’ that makes the subsequent discipline-focused phases of therapy less reliant on punitive force. Similarly, the data support Multisystemic Therapy (MST) frameworks (Hunkin et al., 2025) by highlighting how strengthening the affective family climate can modulate the impact of broader ecological stressors common in the Colombian context. Rather than merely prohibiting violence, these interventions prioritize the enhancement of the affective climate as a core strategy for fostering non-violent disciplinary environments.

The Colombian sociocultural context may further shape this moderation process. In societies where physical punishment has historically been viewed as a legitimate childrearing practice, warmth may coexist with punitive discipline without necessarily being perceived as contradictory (Cuartas et al., 2019b; Lansford et al., 2014). Under these conditions, warmth may retain some buffering capacity, particularly when adolescents interpret disciplinary actions as embedded within an otherwise caring relationship. Nevertheless, as international evidence indicates, this buffering effect is partial and context-dependent, underscoring the need for culturally sensitive interventions. It is also important to highlight that the use of a composite parental warmth score in this study assumes that the family’s affective density functions as a cumulative resource. However, this approach has important conceptual implications, particularly in households where caregivers’ reported warmth differs significantly. In such instances, a moderate composite score may mask a ‘polarized’ affective climate—where one caregiver is highly supportive while the other remains emotionally distant—rather than a ‘moderate’ climate where both caregivers are consistently, though less intensely, warm. From a developmental perspective, these two family structures may place different demands on an adolescent’s emotional regulation. While our aggregate measure effectively captures the ‘net’ emotional safety available to buffer family conflict, it potentially overlooks the specific relational strain caused by parental inconsistency. Future research utilizing dyadic analysis is necessary to determine if the protective value of warmth remains stable when there is high intra-familial discrepancy.

### Implications for Research, Policy, and Intervention

Taken together, these findings highlight the interconnected nature of family processes and reinforce the importance of addressing both relational risk and protective factors in efforts to reduce CP. While family conflict emerges as a robust predictor of CP, parental warmth plays a critical moderating role that can reduce the likelihood that conflict translates into physical discipline.

From a policy perspective, these results provide empirical support for Colombia’s National Pedagogical and Prevention Strategy mandated by Law 2089. Interventions that focus exclusively on prohibiting CP may be insufficient if they fail to address underlying family conflict and emotional regulation. Evidence-based parenting programs that simultaneously promote conflict management skills and strengthen parental warmth are more likely to achieve sustainable reductions in violence (Kaminski & Claussen, 2017).

In conclusion, this study provides empirical evidence of the complex, interplay between risk and protective factors within Colombian family dynamics. The findings confirm that family conflict is a robust and significant positive predictor of the use of CP among adolescents. However, the results also highlight the critical role of parental warmth as a protective buffer. While conflict-ridden environments significantly increase the likelihood of parents resorting to harsh disciplinary measures, the presence of high levels of emotional closeness and support significantly attenuates this association. The high prevalence of CP reported by adolescents underscores the persistent nature of this practice in Colombia, despite recent legal prohibitions under Law 2089 of 2021. Because the protective capacity of warmth is weakened in the face of chronic or destructive conflict, these results suggest that single focus interventions may be insufficient. To effectively reduce the reliance on physical discipline, public policies and clinical interventions must adopt a systemic approach that simultaneously targets the reduction of intrafamilial conflict through conflict resolution training, the enhancement of parental warmth and emotional responsiveness and the promotion of non-violent disciplinary strategies tailored to the specific cultural and socioeconomic realities of the community.

Ultimately, fostering a warm and supportive family environment is not a matter of affective expression: it is a vital relational resource that protects adolescents from the escalating cycle of conflict and punitive discipline. By strengthening these bonds, we can contribute to the fulfillment of national and international mandates to protect the well-being and fundamental rights of children and adolescents.

### Limitations

Several limitations warrant consideration when interpreting these findings. First, the cross-sectional design limits the ability to establish temporal precedence or definitive causal pathways among family conflict, parental warmth, and CP. While the moderation model is theoretically grounded, relationships may be bidirectional, as chronic punishment may exacerbate family tension. Second, the study uses a single-informant design, relying exclusively on adolescent self-reports. This may introduce common methods’ bias and subjective perceptions related to memory or social desirability. Third, the single-item measure for the CP while ethically necessary due to the legal prohibition of CP in Colombia (Law 2089 of 2021) – constrains the reliability and interpretability of the construct by not capturing the severity or specific types of physical discipline. Finally, the sample was drawn from public schools in specific municipalities within anonimized, Colombia, serving primarily low-to-middle socioeconomic strata, which may limit the generalizability of these results to other regional or higher-income contexts in Colombia. Future research should use longitudinal, multi-informant strategies and diversified national samples to further validate these dynamics. Finally, it is important to acknowledge that the use of a composite mean for parental warmth may mask substantial discrepancies between caregivers. Although a high average score represents a significant emotional resource, a household with disparate levels of warmth between parents may present a different developmental environment than one characterized by moderate consistency. Future research using multi-informant strategies and longitudinal designs is needed to further validate these dynamics and explore the impact of parental inconsistency on adolescent adjustment.

## Data Availability

The data that support the findings of this study are available with requests to the corresponding author.
